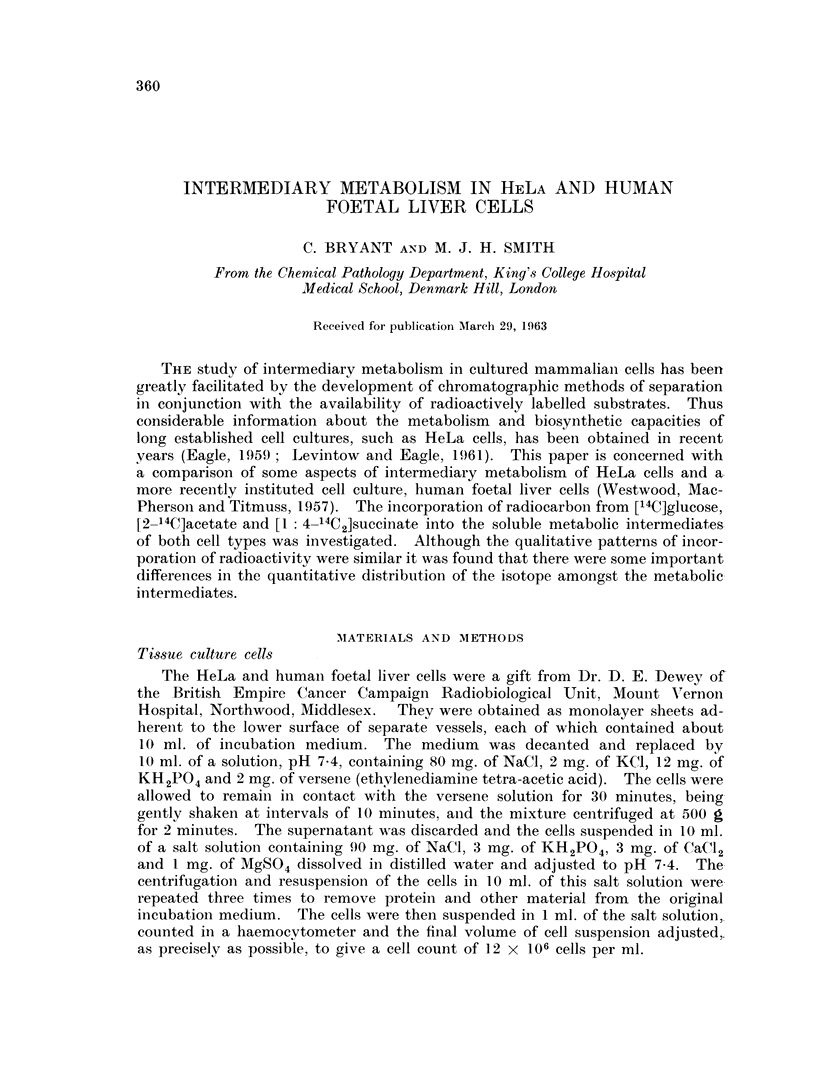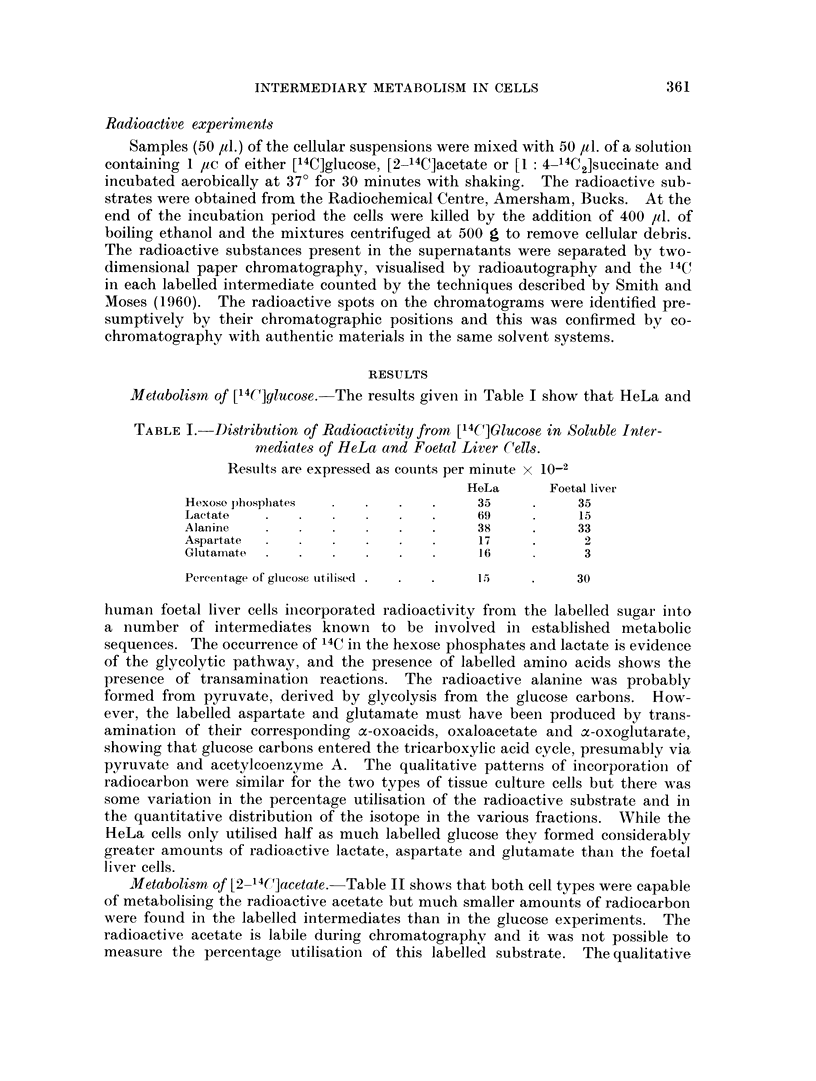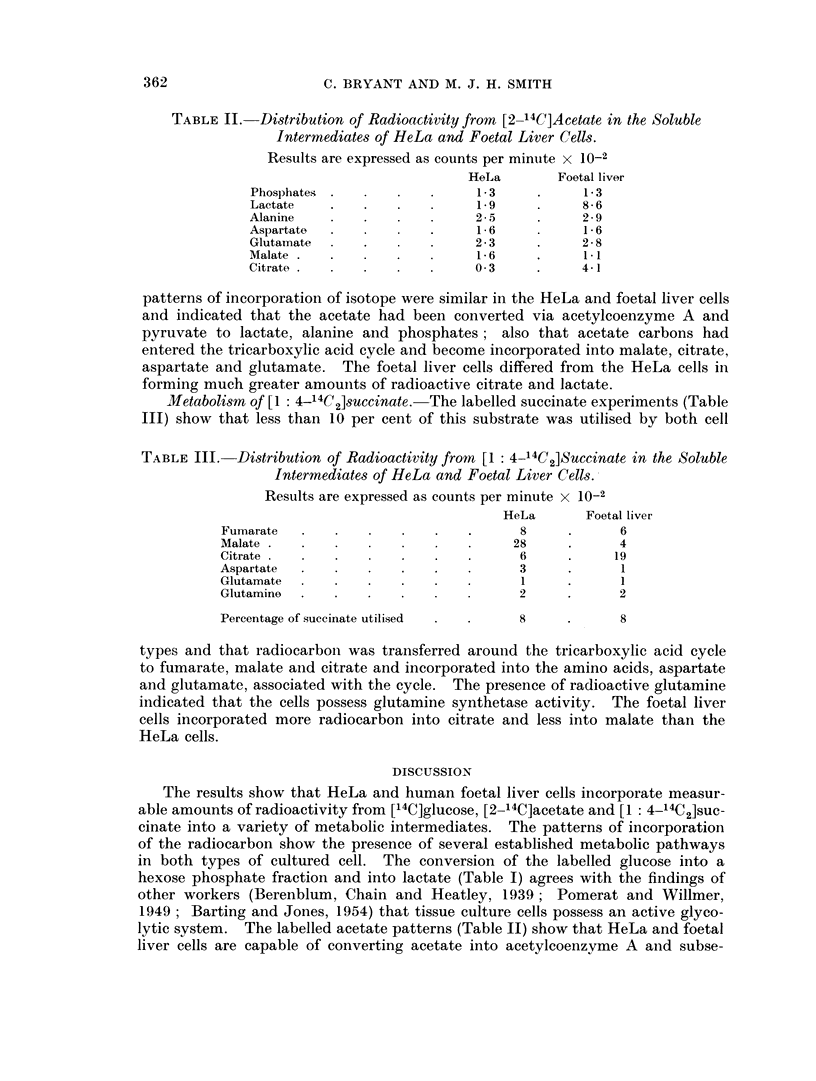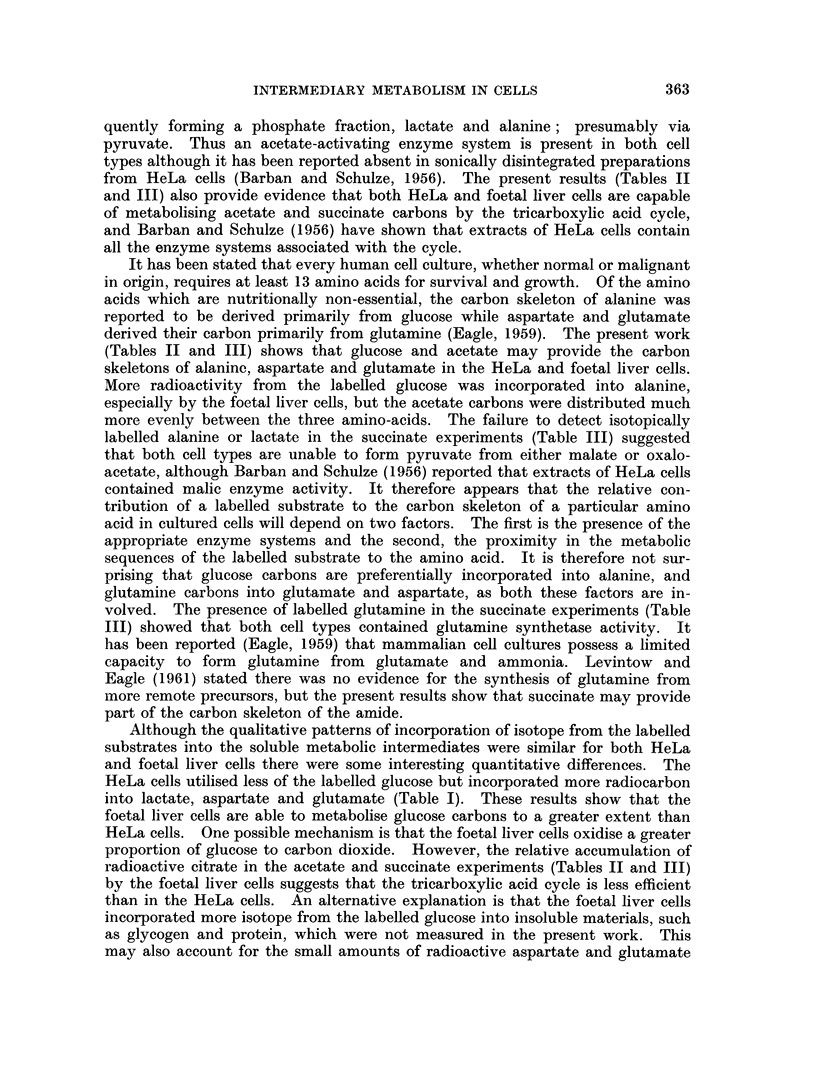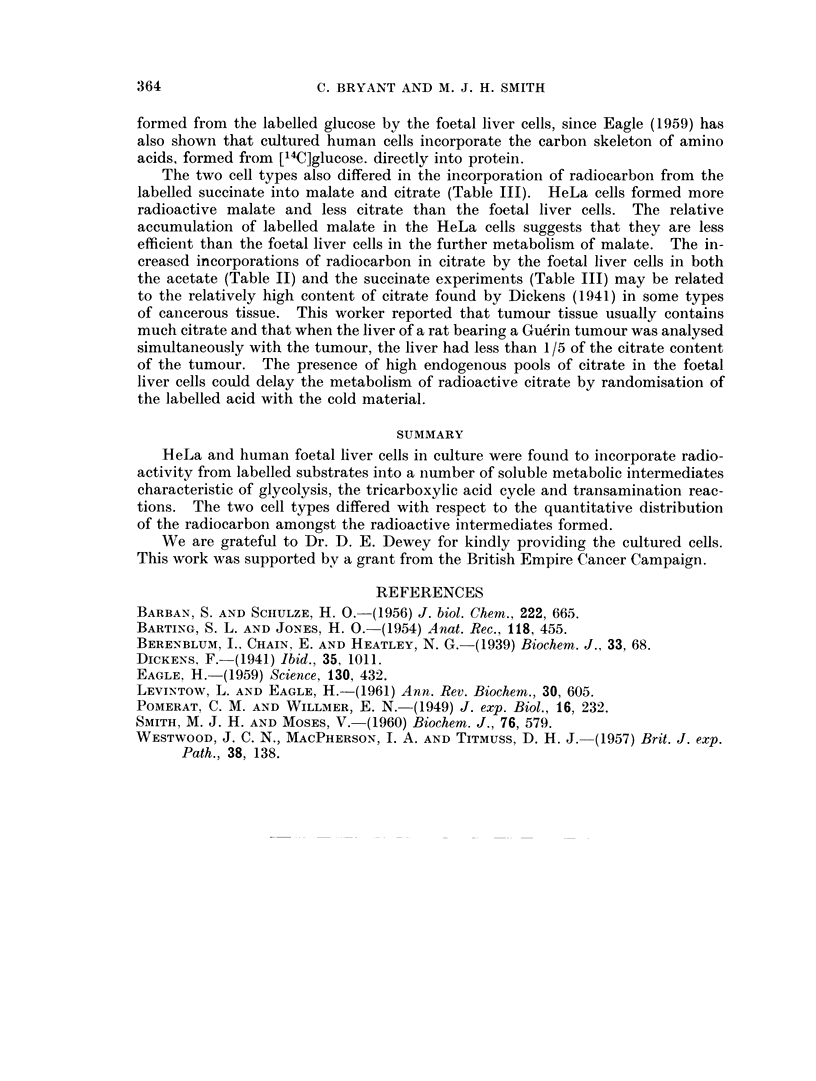# Intermediary Metabolism in HeLa and Human Foetal Liver Cells

**DOI:** 10.1038/bjc.1963.49

**Published:** 1963-06

**Authors:** C. Bryant, M. J. H. Smith


### INTERMEDIARY METABOLISM IN HeLa AND HUMAN FOETAL LIVER CELLS

C. BRYANT AND M. J. H. SMITH

*From the Chemical Pathology Department, King's College Hospital Medical School, Denmark Hill, London*

Received for publication March 29, 1963

THE study of intermediary metabolism in cultured mammalian cells has been greatly facilitated by the development of chromatographic methods of separation in conjunction with the availability of radioactively labelled substrates. Thus considerable information about the metabolism and biosynthetic capacities of long established cell cultures, such as HeLa cells, has been obtained in recent years (Eagle, 1959; Levintow and Eagle, 1961). This paper is concerned with a comparison of some aspects of intermediary metabolism of HeLa cells and a more recently instituted cell culture, human foetal liver cells (Westwood, MacPherson and Titmuss, 1957). The incorporation of radiocarbon from [$^{14}C$]glucose, [2–$^{14}C$]acetate and [1 : 4–$^{14}C_2$]succinate into the soluble metabolic intermediates of both cell types was investigated. Although the qualitative patterns of incorporation of radioactivity were similar it was found that there were some important differences in the quantitative distribution of the isotope amongst the metabolic intermediates.

### MATERIALS AND METHODS

### *Tissue culture cells*

The HeLa and human foetal liver cells were a gift from Dr. D. E. Dewey of the British Empire Cancer Campaign Radiobiological Unit, Mount Vernon Hospital, Northwood, Middlesex. They were obtained as monolayer sheets adherent to the lower surface of separate vessels, each of which contained about 10 ml. of incubation medium. The medium was decanted and replaced by 10 ml. of a solution, pH 7·4, containing 80 mg. of NaCl, 2 mg. of KCl, 12 mg. of $KH_2PO_4$ and 2 mg. of versene (ethylenediamine tetra-acetic acid). The cells were allowed to remain in contact with the versene solution for 30 minutes, being gently shaken at intervals of 10 minutes, and the mixture centrifuged at 500 g for 2 minutes. The supernatant was discarded and the cells suspended in 10 ml. of a salt solution containing 90 mg. of NaCl, 3 mg. of $KH_2PO_4$, 3 mg. of $CaCl_2$ and 1 mg. of $MgSO_4$ dissolved in distilled water and adjusted to pH 7·4. The centrifugation and resuspension of the cells in 10 ml. of this salt solution were repeated three times to remove protein and other material from the original incubation medium. The cells were then suspended in 1 ml. of the salt solution, counted in a haemocytometer and the final volume of cell suspension adjusted, as precisely as possible, to give a cell count of $12 \times 10^6$ cells per ml.

*Radioactive experiments*

Samples (50 $\mu$l.) of the cellular suspensions were mixed with 50 $\mu$l. of a solution containing 1 $\mu$c of either [$^{14}$C]glucose, [2–$^{14}$C]acetate or [1 : 4–$^{14}C_2$]succinate and incubated aerobically at 37° for 30 minutes with shaking. The radioactive substrates were obtained from the Radiochemical Centre, Amersham, Bucks. At the end of the incubation period the cells were killed by the addition of 400 $\mu$l. of boiling ethanol and the mixtures centrifuged at 500 g to remove cellular debris. The radioactive substances present in the supernatants were separated by two-dimensional paper chromatography, visualised by radioautography and the $^{14}$C in each labelled intermediate counted by the techniques described by Smith and Moses (1960). The radioactive spots on the chromatograms were identified presumptively by their chromatographic positions and this was confirmed by co-chromatography with authentic materials in the same solvent systems.

### RESULTS

*Metabolism of* [$^{14}$*C*]*glucose.*—The results given in Table I show that HeLa and

TABLE I.—*Distribution of Radioactivity from* [$^{14}$*C*]*Glucose in Soluble Intermediates of HeLa and Foetal Liver Cells.*

Results are expressed as counts per minute $\times 10^{-2}$

| | HeLa | Foetal liver |
|---|---|---|
| Hexose phosphates | 35 | 35 |
| Lactate | 69 | 15 |
| Alanine | 38 | 33 |
| Aspartate | 17 | 2 |
| Glutamate | 16 | 3 |
| Percentage of glucose utilised | 15 | 30 |

human foetal liver cells incorporated radioactivity from the labelled sugar into a number of intermediates known to be involved in established metabolic sequences. The occurrence of $^{14}$C in the hexose phosphates and lactate is evidence of the glycolytic pathway, and the presence of labelled amino acids shows the presence of transamination reactions. The radioactive alanine was probably formed from pyruvate, derived by glycolysis from the glucose carbons. However, the labelled aspartate and glutamate must have been produced by transamination of their corresponding $\alpha$-oxoacids, oxaloacetate and $\alpha$-oxoglutarate, showing that glucose carbons entered the tricarboxylic acid cycle, presumably via pyruvate and acetylcoenzyme A. The qualitative patterns of incorporation of radiocarbon were similar for the two types of tissue culture cells but there was some variation in the percentage utilisation of the radioactive substrate and in the quantitative distribution of the isotope in the various fractions. While the HeLa cells only utilised half as much labelled glucose they formed considerably greater amounts of radioactive lactate, aspartate and glutamate than the foetal liver cells.

*Metabolism of* [2–$^{14}$*C*]*acetate.*—Table II shows that both cell types were capable of metabolising the radioactive acetate but much smaller amounts of radiocarbon were found in the labelled intermediates than in the glucose experiments. The radioactive acetate is labile during chromatography and it was not possible to measure the percentage utilisation of this labelled substrate. The qualitative

TABLE II.—*Distribution of Radioactivity from [2–$^{14}C$]Acetate in the Soluble Intermediates of HeLa and Foetal Liver Cells.*

Results are expressed as counts per minute $\times$ $10^{-2}$

| | HeLa | Foetal liver |
|---|---|---|
| Phosphates | 1·3 | 1·3 |
| Lactate | 1·9 | 8·6 |
| Alanine | 2·5 | 2·9 |
| Aspartate | 1·6 | 1·6 |
| Glutamate | 2·3 | 2·8 |
| Malate | 1·6 | 1·1 |
| Citrate | 0·3 | 4·1 |

patterns of incorporation of isotope were similar in the HeLa and foetal liver cells and indicated that the acetate had been converted via acetylcoenzyme A and pyruvate to lactate, alanine and phosphates ; also that acetate carbons had entered the tricarboxylic acid cycle and become incorporated into malate, citrate, aspartate and glutamate. The foetal liver cells differed from the HeLa cells in forming much greater amounts of radioactive citrate and lactate.

*Metabolism of* [1 : 4–$^{14}C_2$]*succinate.*—The labelled succinate experiments (Table III) show that less than 10 per cent of this substrate was utilised by both cell

TABLE III.—*Distribution of Radioactivity from* [1 : 4–$^{14}C_2$]*Succinate in the Soluble Intermediates of HeLa and Foetal Liver Cells.*

Results are expressed as counts per minute $\times$ $10^{-2}$

| | HeLa | Foetal liver |
|---|---|---|
| Fumarate | 8 | 6 |
| Malate | 28 | 4 |
| Citrate | 6 | 19 |
| Aspartate | 3 | 1 |
| Glutamate | 1 | 1 |
| Glutamine | 2 | 2 |
| Percentage of succinate utilised | 8 | 8 |

types and that radiocarbon was transferred around the tricarboxylic acid cycle to fumarate, malate and citrate and incorporated into the amino acids, aspartate and glutamate, associated with the cycle. The presence of radioactive glutamine indicated that the cells possess glutamine synthetase activity. The foetal liver cells incorporated more radiocarbon into citrate and less into malate than the HeLa cells.

### DISCUSSION

The results show that HeLa and human foetal liver cells incorporate measurable amounts of radioactivity from [$^{14}$C]glucose, [2–$^{14}$C]acetate and [1 : 4–$^{14}C_2$]succinate into a variety of metabolic intermediates. The patterns of incorporation of the radiocarbon show the presence of several established metabolic pathways in both types of cultured cell. The conversion of the labelled glucose into a hexose phosphate fraction and into lactate (Table I) agrees with the findings of other workers (Berenblum, Chain and Heatley, 1939 ; Pomerat and Willmer, 1949 ; Barting and Jones, 1954) that tissue culture cells possess an active glycolytic system. The labelled acetate patterns (Table II) show that HeLa and foetal liver cells are capable of converting acetate into acetylcoenzyme A and subsequently forming a phosphate fraction, lactate and alanine ; presumably via pyruvate. Thus an acetate-activating enzyme system is present in both cell types although it has been reported absent in sonically disintegrated preparations from HeLa cells (Barban and Schulze, 1956). The present results (Tables II and III) also provide evidence that both HeLa and foetal liver cells are capable of metabolising acetate and succinate carbons by the tricarboxylic acid cycle, and Barban and Schulze (1956) have shown that extracts of HeLa cells contain all the enzyme systems associated with the cycle.

It has been stated that every human cell culture, whether normal or malignant in origin, requires at least 13 amino acids for survival and growth. Of the amino acids which are nutritionally non-essential, the carbon skeleton of alanine was reported to be derived primarily from glucose while aspartate and glutamate derived their carbon primarily from glutamine (Eagle, 1959). The present work (Tables II and III) shows that glucose and acetate may provide the carbon skeletons of alanine, aspartate and glutamate in the HeLa and foetal liver cells. More radioactivity from the labelled glucose was incorporated into alanine, especially by the foetal liver cells, but the acetate carbons were distributed much more evenly between the three amino-acids. The failure to detect isotopically labelled alanine or lactate in the succinate experiments (Table III) suggested that both cell types are unable to form pyruvate from either malate or oxalo-acetate, although Barban and Schulze (1956) reported that extracts of HeLa cells contained malic enzyme activity. It therefore appears that the relative contribution of a labelled substrate to the carbon skeleton of a particular amino acid in cultured cells will depend on two factors. The first is the presence of the appropriate enzyme systems and the second, the proximity in the metabolic sequences of the labelled substrate to the amino acid. It is therefore not surprising that glucose carbons are preferentially incorporated into alanine, and glutamine carbons into glutamate and aspartate, as both these factors are involved. The presence of labelled glutamine in the succinate experiments (Table III) showed that both cell types contained glutamine synthetase activity. It has been reported (Eagle, 1959) that mammalian cell cultures possess a limited capacity to form glutamine from glutamate and ammonia. Levintow and Eagle (1961) stated there was no evidence for the synthesis of glutamine from more remote precursors, but the present results show that succinate may provide part of the carbon skeleton of the amide.

Although the qualitative patterns of incorporation of isotope from the labelled substrates into the soluble metabolic intermediates were similar for both HeLa and foetal liver cells there were some interesting quantitative differences. The HeLa cells utilised less of the labelled glucose but incorporated more radiocarbon into lactate, aspartate and glutamate (Table I). These results show that the foetal liver cells are able to metabolise glucose carbons to a greater extent than HeLa cells. One possible mechanism is that the foetal liver cells oxidise a greater proportion of glucose to carbon dioxide. However, the relative accumulation of radioactive citrate in the acetate and succinate experiments (Tables II and III) by the foetal liver cells suggests that the tricarboxylic acid cycle is less efficient than in the HeLa cells. An alternative explanation is that the foetal liver cells incorporated more isotope from the labelled glucose into insoluble materials, such as glycogen and protein, which were not measured in the present work. This may also account for the small amounts of radioactive aspartate and glutamate formed from the labelled glucose by the foetal liver cells, since Eagle (1959) has also shown that cultured human cells incorporate the carbon skeleton of amino acids, formed from [$^{14}$C]glucose, directly into protein.

The two cell types also differed in the incorporation of radiocarbon from the labelled succinate into malate and citrate (Table III). HeLa cells formed more radioactive malate and less citrate than the foetal liver cells. The relative accumulation of labelled malate in the HeLa cells suggests that they are less efficient than the foetal liver cells in the further metabolism of malate. The increased incorporations of radiocarbon in citrate by the foetal liver cells in both the acetate (Table II) and the succinate experiments (Table III) may be related to the relatively high content of citrate found by Dickens (1941) in some types of cancerous tissue. This worker reported that tumour tissue usually contains much citrate and that when the liver of a rat bearing a Guérin tumour was analysed simultaneously with the tumour, the liver had less than 1/5 of the citrate content of the tumour. The presence of high endogenous pools of citrate in the foetal liver cells could delay the metabolism of radioactive citrate by randomisation of the labelled acid with the cold material.

### SUMMARY

HeLa and human foetal liver cells in culture were found to incorporate radioactivity from labelled substrates into a number of soluble metabolic intermediates characteristic of glycolysis, the tricarboxylic acid cycle and transamination reactions. The two cell types differed with respect to the quantitative distribution of the radiocarbon amongst the radioactive intermediates formed.

We are grateful to Dr. D. E. Dewey for kindly providing the cultured cells. This work was supported by a grant from the British Empire Cancer Campaign.

### REFERENCES

Barban, S. and Schulze, H. O.—(1956) *J. biol. Chem.*, **222**, 665.
Barting, S. L. and Jones, H. O.—(1954) *Anat. Rec.*, **118**, 455.
Berenblum, I., Chain, E. and Heatley, N. G.—(1939) *Biochem. J.*, **33**, 68.
Dickens, F.—(1941) *Ibid.*, **35**, 1011.
Eagle, H.—(1959) *Science*, **130**, 432.
Levintow, L. and Eagle, H.—(1961) *Ann. Rev. Biochem.*, **30**, 605.
Pomerat, C. M. and Willmer, E. N.—(1949) *J. exp. Biol.*, **16**, 232.
Smith, M. J. H. and Moses, V.—(1960) *Biochem. J.*, **76**, 579.
Westwood, J. C. N., MacPherson, I. A. and Titmuss, D. H. J.—(1957) *Brit. J. exp. Path.*, **38**, 138.